# Antioxidant and Anti-Inflammatory Properties of an Extract Rich in Polysaccharides of the Mushroom *Polyporus dermoporus*

**DOI:** 10.3390/antiox3040730

**Published:** 2014-11-04

**Authors:** Celina Maria P. Guerra Dore, Monique Gabriela das Chagas F. Alves, Maria da Glória L. Santos, Leonardo Augusto R. de Souza, Iuri Goulart Baseia, Edda Lisboa Leite

**Affiliations:** 1 Laboratory of Glycobiology, Department of Biochemistry, Federal University of Rio Grande do Norte, Av. Salgado Filho, 3000, Bairro L. Nova, CEP 59072-940, Natal, Brazil; E-Mails: cmpguerra@hotmail.com (C.M.P.G.D.); moniquegabi@gmail.com (M.G.C.F.A.); gloria_bios@hotmail.com (M.G.L.S.); leo_augustopdf@hotmail.com (L.A.R.S.); 2 Laboratory of Mycology, Department of Botany, Ecology and Zoology, Federal University of Rio Grande do Norte, Av. Salgado Filho, 3000, Bairro L. Nova 59078-970, Natal, Brazil; E-Mail: baseia@cb.ufrn.br

**Keywords:** antioxidant activities, mushroom, anti-inflammatory effect, glucan-protein, *Polyporus dermoporus*, polysaccharides

## Abstract

*Polyporus dermoporus* mushroom, native to Brazil, is produced under natural conditions in the unexplored reserve of Mata da Estrela-Rio Grande do Norte-RN. These mushrooms were delipidated with chloroform:methanol (2:1 v/v), extracted with water at 100 °C, and fractionated with ethanol (one and three volumes) and then centrifuged. The ethanol precipitation showed a high total sugar level of 64.8% and 1% of protein. This precipitate contained a high glucan level, characterized by chemical methods and by NMR of ^13^C and ^1^H and spectroscopy. The ^13^C NMR spectrum of these mushroom extracts showed the presence of β-glucose by a signal at 103.25 ppm. Studies with these glucans were made to elucidate antioxidant and anti-inflammatory activities. This extract of glucans inhibited the lipid peroxidation (42.9%) and superoxide radicals (83.3%) at 67 μg/mL. However, the inhibition of hydroxyl radical by the extract of this mushroom was 96% at 267 μg/mL. The action of this extract on induced pleurisy showed a 92.5% and 68.7% reduction in polymorphonuclears cells and nitric oxide, respectively, at 30 mg/kg. The glucans reduced the croton oil-induced ear edema by 65.6% at 30 mg/kg.

## 1. Introduction

Fungal polysaccharides have recently received considerable attention, due to their potential use in a wide variety of industries, including cosmetics, pharmaceuticals and food. [1,2,3]. Several polysaccharides and polysaccharide-protein complexes have been isolated from fungi (mushrooms) and are being used as a source of therapeutic agents [2,3]. Many, if not all, basidiomycete mushrooms contain biologically active polysaccharides in fruit bodies, culture mycelium and culture broth [1,4].

Oxidation is essential to many living organisms for the production of energy to fuel biological processes. Free radicals are produced in normal and/or pathological cell metabolism [5]. However, the uncontrolled production of oxygen-derived free radicals is involved in the onset of many diseases, such as cancer, rheumatoid arthritis, cirrhosis and arteriosclerosis, as well as in degenerative processes associated with aging [5,6]. It has long been recognized that many naturally occurring substances in plants and fungi have antioxidant activities. Several species of mushroom contain a great variety of molecules, scavenger-free radicals or reactive oxygen species, such as polysaccharides and phenolic compounds [6,7,8,9]. This explains why mushrooms have recently become attractive as nutritionally beneficial foods and as a source material for the development of drugs. Several types of inflammatory tissue injury are mediated by reactive oxygen metabolites. The most likely sources of these oxidizing agents are the phagocytic leukocytes (e.g., neutrophils, monocytes, macrophages and eosinophils) that invade the tissue. It is becoming increasingly apparent that in addition to promoting cytotoxicity, reactive oxygen metabolites may also initiate and/or increase inflammation [6,10].

Recent studies have shown that a glucan-protein complex from the fungus *Geastrum saccatum* has strong anti-inflammatory and antioxidant activity [6,7]. Studies with polysaccharides from the mushroom *Polyporus albicans* showed that these compounds have powerful stimulating effects on murine lymphocyte proliferation induced by concanavalin A or lipopolysaccharides and that its branches are extremely important for enhancement expression [8].

In this work, chemical features of a β-glucan-protein complex from *P. dermoporus*, as well as its anti-inflammatory antioxidant and cytotoxic activities were analyzed.

## 2. Experimental Section

### 2.1. Mushroom

Fruiting bodies of fungi *Polyporus dermoporus* Pers. (Polyporaceae) were obtained from a private reserve, Mata da Estrela, of the Atlantic Forest in the state of Rio Grande do Norte (06°22′25″ S, 35°01′24″ W) Brazil in July, 2009. The mushroom was identified by Prof. Iuri Goulart Baseia, Department of Botany, Ecology and Zoology of Universidade Federal do Rio Grande do Norte (UFRN), Natal, Brazil. A voucher specimen was deposited in the herbarium of this department.

### 2.2. Materials

Thiobarbituric acid, D_2_O, sodium salicylate, nitroblue tetrazolium, phenazine methosulfate, EDTA, H_2_O_2,_ indomethacin, croton oil, NADH, Ficoll isopaque, carrageenan, Folin Ciocalteu, gallic acid, ketamine, xylazine and mercaptoethanol were purchased from Sigma Aldrich (St. Louis, MO, USA). RPMI 1640 medium was purchased from Gibco (Grand Island, NY, USA). All other chemicals used were of analytical grade.

### 2.3. Animals

Male BALBc mice (about 25 g) and male Wistar rats (150–180 g) were housed in temperature-controlled rooms (22–23 °C) until use. Animals were given *ad libitum* access to food and water. Each experimental group contained 6 animals. The mice were used in the croton oil-induced ear edema test and the rat in pleurisy. All of the experiments were performed in accordance with the guidelines and regulations set forth by the Ethics Committee, Centro de Biociencias, UFRN.

### 2.4. Extract Preparation

Fruiting bodies of *Polyporus dermoporus* were dried at 40 °C and powdered. The resulting powder was delipidated with ether and placed in solution in a proportion of 10 g of powder to 100 mL of distilled water at 100 °C for 3 h. Composition analysis of this extract was made [11]. Then, 1 volume of ethanol (50 mL) was added to the supernatant (50 mL). Subsequently, the extract was centrifuged, and 3 volumes of ethanol (150 mL) were added. The precipitate, rich in polysaccharides, was evaporated in a vacuum to obtain a solid extract.

### 2.5. Chemical Analysis

The total protein of the extract was determined by Spector’s method [12]. Total sugar content was determined by the phenol sulfuric acid colorimetric method for the determination of total sugars (the Dubois method) [13].

### 2.6. ^13^C nuclear Magnetic Resonance Spectroscopy (NMR)

^13^C and 1H NMR spectroscopy experiments were conducted using a 200-MHz Varian Mercury 200 Magneto Oxford spectrometer at 60 °C, and the sample was dissolved in deuterated water (D_2_O).

### 2.7. *In Vitro* Antioxidant Tests

#### 2.7.1. Superoxide Radicals

Superoxide radicals were generated in 3 mL Tris-HCl buffer (16 mM, pH 8.0), which contained 78 μM NADH (reduced form), 50 μM nitroblue tetrazolium, 10 μM phenazine methosulfate [14] and varying concentrations of polysaccharides (67–267 μg/mL). The color reaction of superoxide radical and nitroblue tetrazolium was detected by monitoring the absorbance at 560 nm. In the essential control, NADH was substituted for Tris-HCl buffer [14]. The percentage of scavenging activity on superoxide radicals was determined by this formula: (%) = [(A_positive control_ − A_sample_)/A_positive control_ − A_negative control_)] × 100; where A_positive control_ is positive control absorbance (all reagents without sample), A_sample_ is sample absorbance and A_negative_ is negative control absorbance (NADH absence).

#### 2.7.2. Hydroxyl Radical Assay

Hydroxyl radicals were generated by an innovation of the Smirnoff and Cumbes 1989 method [15] in sodium phosphate buffer (150 mM, pH 7.4), which contained 0.15 mM FeSO_4_-EDTA, 2 mM sodium salicylate, 6 mM H_2_O_2_ and varying concentrations of polysaccharides (67–267 μg/mL). In the essential control, sodium phosphate buffer replaced H_2_O_2_. The solutions were incubated at 37 °C for 1 h and detected by monitoring the absorbance at 510 nm. The inhibition rate (%) = 1 − (Abs_1_/Abs_2_) × 100%, where Abs_1_ is the absorbance obtained from the reaction with the samples and Abs_2_ is the absorbance of the reaction in which distilled water replaced hydrogen peroxide.

#### 2.7.3. Lipid Peroxides Radical Scavenging Capacity

Liver microsomes were prepared from male Wistar rats according to the method proposed by [16]. The protein content of the microsomes was measured by Spector’s method [12]. The product of microsomal lipid peroxidation was malondialdehyde. Microsomes (200–300 μg/mL) were incubated at 37 °C for 1 h with varying amounts of polysaccharides (67–267 μg/mL), 10 μM FeSO_4_ and 0.1 mM ascorbic acid in 1.0 mL potassium phosphate buffer solution (0.2 M, pH 7.4). The reaction was stopped by 20% trichloroacetic acid (1.0 mL) and 0.67% 2-thiobarbituric acid (1.5 mL) in succession, and the solution was then heated at 100 °C for 15 min. After precipitation, the proteins were removed by centrifugation. The color reaction of the malondialdehyde-thiobarbituric acid complex was detected by monitoring the absorbance at 532 nm [16]. The essential control did not contain FeSO_4_ or ascorbic acid. The percentage of antioxidant activity of the samples was evaluated according to the following formula: inhibition rate (%) = (A_0_ − A)/(A_0_ − A_e_) × 100%; where A_0_ is the absorbance of the free radical generation system, A is the absorbance of the test sample and A_e_ is the absorbance of the essential control.

### 2.8. Assessment Phenolic Compounds

Phenolic compounds extract from *P. dermoporus* were measured by the Folin Ciocalteu method [17] with few modifications using 0.5 mL ethanol, 2.5 mL distilled water, 0.25 mL Folin Ciocalteu reagent, 0.5 mL sodium carbonate and 0.5 mL of polysaccharides (1 mg/mL). Absorbance was determined at 765 nm against the blank, and a gallic acid calibration curve (0–500 mg/mL) was constructed and used to determine the total phenolic content of the samples, which were expressed as gallic acid equivalents (GAE).

### 2.9. Croton Oil-Induced Ear Edema Test

The croton oil-induced ear edema test was performed as previously described [18,19]. The animals were anesthetized with 80 mg**/**kg ketamine and 12 mg**/**kg xylazine (3:1, i.p.). A total of 20 μL (0.4 μg of croton oil in acetone) was applied to the inner surface of the right ear of each mouse (subcutaneous). After 24 h, the extract, rich in polysaccharide, was intravenously administered at doses of 10, 30 and 50 mg/kg by body weight. The left ear remained untreated. Control animals only received the irritant. The animals were killed by cervical dislocation 24 h after the treatment was removed from both the treated and untreated ear. The difference in the thickness between the two plugs before and after the treatment was measured to determine the edematous response.

### 2.10. Extract Action in Carrageenan-Induced Pleurisy

The animals were anesthetized intraperitoneally with 20 mg/mL solution of xylazine hydrochloride and a 50 mg/mL solution of ketamine hydrochloride at a 1:2 proportion of 100 mg/kg/weight. The animals had been previously treated with 10, 30 and 50 mg/kg i.p. by body weight of the extract, with the exception of the control group animals (carrageenan (control+) and saline (control−)). After 30 min, the animals were submitted to a skin incision at the sixth intercostal space level. The muscles were dissected, and 0.2 mL of saline containing 1% (w/v) carrageenan were injected into the pleural cavity. The skin incision was closed with a suture, and the animals were allowed to recover. The animals were killed 4 h after carrageenan injection. The pleural cavity was carefully opened and washed with 2 mL of saline solution containing heparin (5 U/mL) and diclofenac (10 μg/mL). The exudate was removed by aspiration. The number of leukocytes for the differential leucocyte count (DLC) in the exudates was counted with an optical microscope after Türk’s staining.

#### Measurement of Nitrite-Nitrate Concentration in Pleural Exudates

Total nitrite in exudates, an indicator of NO synthesis, was measured as previously described by Cuzzocrea *et al.* 1998 [19]. Briefly, the nitrate in the samples was first reduced to nitrite by incubation with nitrate reductase (670 mU/mL) and NADPH (160 μM) at room temperature for 3 h. The total nitrite concentration in the samples was then measured using the Griess reaction, by adding 100 μL of Griess reagent ((0.1% (w/v) naphthylethylenediamide dihydrochloride in H_2_O and 1% (w/v) sulfanilamide in 5% (v/v) concentrated H_3_PO_4_(vol 1:1)) to the 100 μL sample [20]. The total nitrite concentration in the samples was then measured using the Griess reaction, by adding 100 μL of Griess reagent. The concentrations were calculated by comparison with the OD_550_ of standard solutions of sodium nitrite prepared in H_2_O.

### 2.11. Histological Examination

For histological examination, ear biopsies were taken 48 h after edema induction by croton oil injection. The tissue slices were fixed in 10% neutral-buffered formaldehyde, embedded in paraffin and sectioned. The sections were stained with hematoxylin and eosin.

### 2.12. Colorimetric MTT (tetrazolium) Assay

The cytotoxicity of the extract was measured as previously described by Mosmann, 1983 [21]. The leukocytes were obtained from human peripheral blood, previously added with heparin, and centrifuged with Ficoll isopaque (Histopaque-1077). The cells were washed successively with RPMI 1640 medium and supplemented with 50 μM 2-mercaptoethanol and 5%–10% fetal bovine serum, in a 6% CO_2_ atmosphere. A total of 100 μL of the solution with 1 × 10^6^ cells/well was added. These cells were incubated for 4 h, and different concentrations (0.5, 1.0 and 1.5 μg/mL) of the extract from *P. dermoporus* and stock MTT solution (10 μL/100 μL medium RPMI) were added to the wells. The plates were incubated at 37 °C for 4 h. Acid-isopropanol (100 μL of 0.04 N HCl) was added to all of the wells and mixed thoroughly to dissolve the dark blue crystals. The plates were read by a MicroElisa reader at 570 nm.

### 2.13. Statistical Analysis

Values are expressed as the mean ± SEM. Analysis of variance (ANOVA, Tukey–Kramer test) was used to evaluate the data, and *p* < 0.05 was accepted as statistically significant. The statistical analysis involved the use of the software, GraphPad Prism 5 (GraphPad Software Inc., San Diego, CA, USA).

## 3. Results and Discussion

### 3.1. Chemical Analysis

In this work, we investigated the chemical composition of the tissue of this native mushroom. The polysaccharides resulting from the extraction of *Polyporus dermoporus* and their cytotoxic, antioxidant, and anti-inflammatory action were studied. The results of the analyses of the composition of the tissue showed levels of carbohydrates, proteins, lipids and ash of 51.8%, 21.4%, 1.66% and 10.8%, respectively; assessment by Association of Official Analytical Chemists (A.O.A.C.), 1984 [11]. When submitted to the fractionation with ethanol, the extracts of this mushroom showed 64.8% carbohydrates and 10.0% proteins. Thus, the polysaccharide extract was composed mainly of glucose polymer (64.6%) (see Table 1), which was confirmed by total sugars by the phenol-H_2_SO_4_ reaction using d-glucose as the standard [13]. Phenolic compounds have been shown to possess potent antioxidant properties. Several studies have established links between the consumption of foods containing high concentrations of phenolic and antioxidant compounds and a lower incidence of cardiovascular diseases and cancer [5]. However, in extracts of *Polyporus dermoporus*, the antioxidant properties are not attributed to these bioactive compounds, due to the very low content of phenolic compounds and proteins (Table 1).

### 3.2. ^1^H and ^13^C NMR Spectroscopies of the Extract from Polyporus Dermoporus

**Figure 1:**
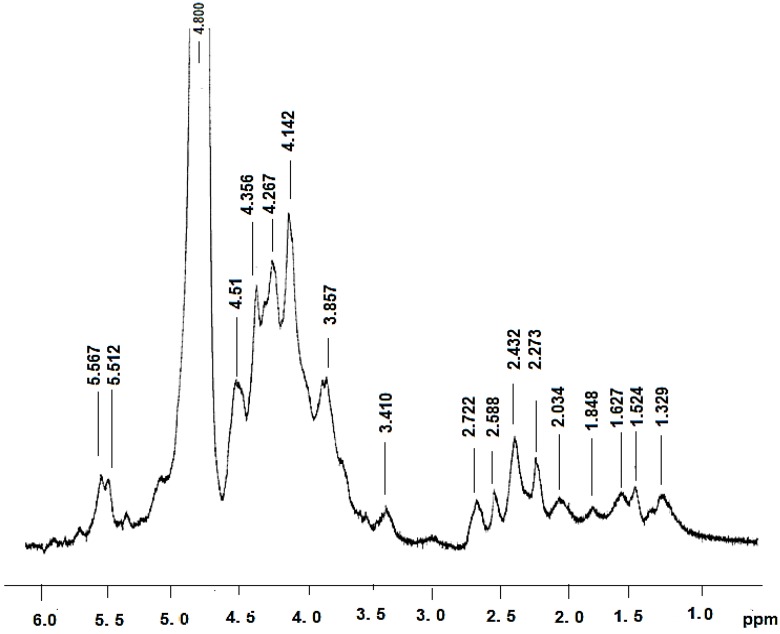
^1^ H NMR spectroscopy of *Polyporus dermoporus* glucan protein.

The ^1^H spectra showed a chemical shift in the anomeric region at 4–6 ppm (Figure 1). In this spectrum, signals 4.1 and 4.6 corresponded to the signals obtained for β glucan [22]. According to Chaveau *et al.*, 1996 [23], the chemical shifts correspond to C2 and C6 [7]. We also observed that the regions between 1.4 and 2.5 are related to the glucan-protein structure. These observations are in accord with other studies [6,7].

**Table 1:** Chemical composition of natural tissue of mushroom *P. dermoporus* (g/100 g dry weight) and of the polysaccharides and proteins from the extract of this mushroom.

Components	%
**Tissue**	
Carbohydrates	51.3 ± 1.32
Proteins	21.4 ± 2.11
Moisture	10.01 ± 1.03
Lipids	1.66 ± 0.51
Ashes	10.80 ± 1.81
**Extract**	
Polysaccharides	64.8 ± 5.23
Proteins	10.2 ± 0.30
Phenolic compounds	0.90 ± 0.02

**Figure 2:**
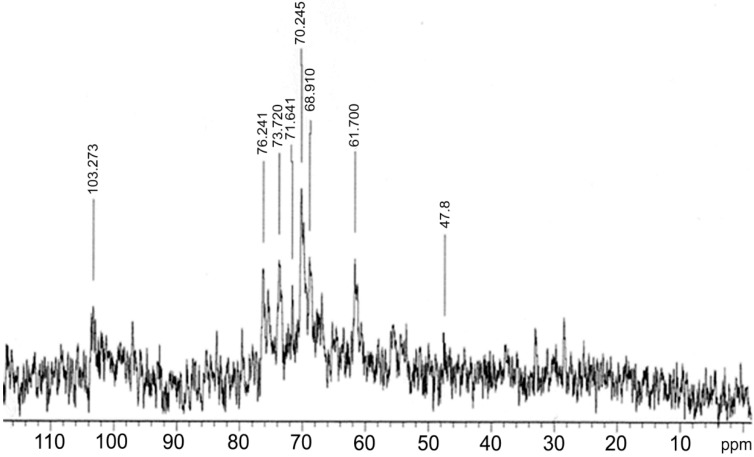
^13^ C NMR spectroscopy of *Polyporus dermoporus* glucan-protein.

The analysis of ^13^C NMR spectroscopy showed that the polysaccharides o this mushroom contain a high level of glucose. This fact is due to the type of homopolysaccharides (glucans) present in this organism. Figure 2 shows the NMR spectra of ^13^C of the extracts from *Polyporus dermoporus.* The presence of glucose can be observed by a signal at 103.2 ppm, characteristic of the β configuration [22,23,24]. Other important signals in the spectra are those in the 60–80 ppm range, where they are related to C2 (73.9 ppm), C3 (84.0 ppm), C4 (80.0 ppm), C5 (76.24 ppm) and C6 (61.7 ppm) of that carbohydrate [25,26,27,28]. A peculiar characteristic of the spectra of β-(1→3) glucans is the presence of at least six signals of similar magnitude (110–60 ppm). The 47.8 ppm signal is related to the –CH_2_N group of amino acids.

### 3.3. Superoxide and Hydroxyl Radicals

Glucans are homopolymers of d-glucose, widely distributed in nature, which may have bioactive properties in various organisms [27,28]. As such, they are highlighted for having a direct influence on the stimulation of phagocytic activity, antitumor properties, antioxidant, immunomodulatory and anti-inflammatory activities [10,26]. The antioxidant effect of the carbohydrates did not correlate with the type of intra-chain linkage, molecular weight or degree of polymer branching. The activity did appear to correlate with the monosaccharide composition of the polymer.

The antioxidant effect did appear to have a relation with the monosaccharide composition of the polymer and the composition of the polymer. Korzaski *et al.*, 2011, in studies with mushroom polysaccharides of the species *A. bisporus*, *A. brasiliensis*, *G. lucidum* and *P. linteus*, showed that scavenging ability is a correlation that was found between the EC_50_ values of the chelating and reducing power abilities and the amount of total glucan content in the extracts [25]. The superoxide radicals were generated in a system of NADH-phenazine-methosulfate with different concentrations of the tested polysaccharides; see Zhou and Zheng *et al.*, 1991 [29].

The formation of hydroxyl radicals (•OH) in the biological systems does not occur enzymatically and which need the presence of iron or transition metals [25]. The removing of •OH is important for the protection of living systems. The results obtained (•OH) were expressed as the % inhibition rate; see Table 2. At 267 μg/mL, 96% of the scavenging activity on hydroxyl radicals was exhibited. This shows a high inhibition in the generation of hydroxyl radicals when compared with polysaccharide from *C. montagnei* (38% at 1000 mg) [11], indicating considerable antioxidant activity in this study.

### 3.4. Determination of Lipid Peroxidation or Malonaldehyde Formation (LPO)

Combined with the generating system of lipid peroxidation using thiobarbituric acid (TBA), a condensation reaction produces malondialdehyde (MDA)-TBA with a pink coloration that can be subsequently quantified [26,30]. The extract of the mushroom used in this work showed efficient inhibition of lipid peroxidation, varying from 24.3% to 42.91% (Table 2). The process of lipid peroxidation results in the formation and propagation of lipid peroxides that act on the cell membrane and microsomes, producing compounds, such as malondialdehyde (MDA) [30,31]. The maximal effect on inhibition microsomal peroxidation occurred at a dose of 67 μg/μL (42.9%). We observed that the inhibition of superoxide radical and peroxidation of this polysaccharide-protein complex is not dose dependent. Polysaccharide of *C. montagnei* at 4 mg/mL produced lipidic peroxidation inhibition of 37.6% ± 0.16%, equivalent to 47% of the activity from α-tocopherol at the same concentration [10].

**Table 2:** The effect of mushroom polysaccharide extracts from *P. dermoporus* on superoxide and hydroxyl radical generation and microsomal lipid peroxidation.

Concentration (μg/mL)	Inhibition Superoxide Radical (%)	Inhibition Hydroxyl Radical (%)	Inhibition Microsomal Peroxidation (%)
67	83.3	20.0	42.9
113	75.1	37.0	40.3
200	51.5	75.0	31.0
267	48.5	96.0	24.3

Each value represents the mean ± S.E.M. (*n* = 6).

Polysaccharides may be acting similarly to other mechanisms described for chelating, by reducing the redox pro-oxidant potential of metals and stabilizing the oxidized form of the metal, through steric interaction and also by forming soluble and stable complexes with metals, which are eventually excreted in the urine, as related by Ebrahimzadeh *et al.* 2008 [9]. This is strengthened by the description of the effect of other polysaccharides in the chelation of metal ions [10,29]. This may explain the strong inhibition of the peroxyl radical when observed in incubation in the presence of glucans, in addition to its significant effect in the reduction of lipid peroxidation, showing its strong antioxidant effect at low concentrations. This can result in protection against the development of pathologies, such as inflammation, cancer, oxidative stress, and diabetes.

### 3.5. Action of the Polysaccharides in Carrageenan-Induced Pleurisy

**Figure 3:**
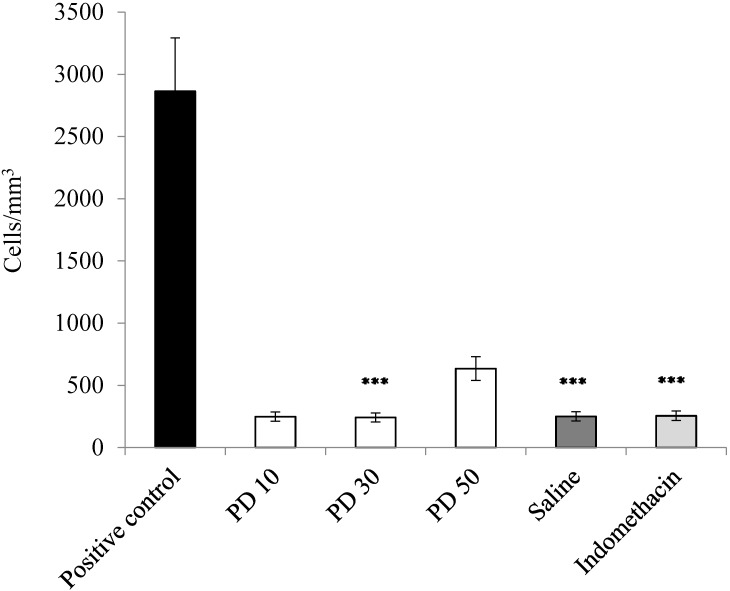
Anti-inflammatory effect of *P. dermoporus* extract (PD) on carrageenan-induced pleurisy. The number of pleural exudate leukocytes in carrageenan-induced Wistar rats. The experimental animals were treated with *P. dermoporus* extract at 10 mg/kg (PD 10), 30 mg/kg (PD 30) and 50 mg/kg (PD 50). Data obtained from animal experiments are expressed as the mean ± SD. The differences between treatment and control were tested by ANOVA. A value of (***) *p* < 0.001 was considered statistically significant.

The results obtained showed a high number of leukocytes cells (2854 cell/mm^3^) in the pleural infiltrate of the positive control (animals treated with carrageenan). When the animals were submitted to the action of carrageenan and treated previously with the glucans of *P. dermoporus* (10 and 30 mg/kg by body weight), the cells obtained in the pleural infiltrate were 681 and 264 cell/mm^3^, respectively (Figure 3). This value corresponds to a reduction of 76% and 92.5% in the number of cells, respectively. At a dose of 50 mg/kg, a 70.6% reduction in infiltrate cells occurs (*p* < 0.001). The nonsteroidal anti-inflammatory drugs (NSAIDs) used in this experimental was indomethacin; *p* < 0.001. ANOVA and the Tukey-Kramer test showed that the difference observed in relation to the control group is highly significant; *p* < 0.001.

### 3.6. Effect of Polysaccharide on Nitric Oxide (NO)

**Figure 4:**
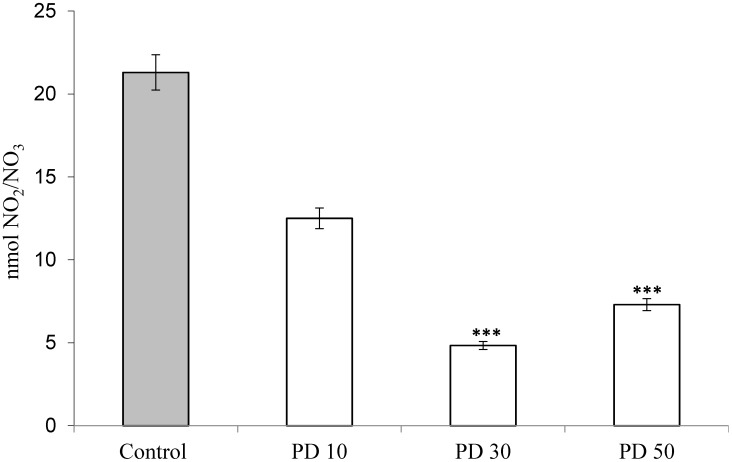
Effect of *P. dermoporus* polysaccharides on NO production from the pleural exudate of Wistar rats with carrageenan-induced pleurisy. The animals were treated with 10 mg/kg (PD 10), 30 mg/kg (PD 30) and 50 mg/kg (PD 50) of *P. dermoporus* extract. Control: Wistar rats with carrageenan-induced pleurisy. Data obtained from animal experiments (*n* = 7) are expressed as the mean ± SD. The differences between the treatment and control were tested using ANOVA. A value of (***) *p* < 0.001 was considered statistically significant.

Polysaccharides derived from mushrooms have been considered an important class of bioactive compounds, and their anti-inflammatory potential has been widely studied [25,26,32]. Nitric oxide in most body fluids is rapidly metabolized to stable products, such as nitrite and nitrate. Figure 4 shows the value of nitrate/nitrite produced after carrageenan administration and previous treatments with the extracts of *P. dermoporus* at doses of 10 mg/kg (PD 10), 30 mg/kg (PD 30) and 50 mg/kg by body weight (PD 50). The positive control (animals with carrageenan) showed 21.39 ± 1.79 nmol of nitrate/nitrite. In the animals that received *P. dermoporus* (30 mg/kg by body weight), this value was 7.48 ± 0.44 nmol (*p* < 0.001). We observed that at 10 mg/kg and 50 mg/kg, the results were 12.51 ± 0.81 and 7.32 ± 1.36 nmol of nitrate/nitrite (*p* < 0.001). In this study, we found the effect of this extract in the modulation of nitric oxide expression in the inflammation model tested.

### 3.7. Croton Oil-Induced Ear Edema Test

**Figure 5:**
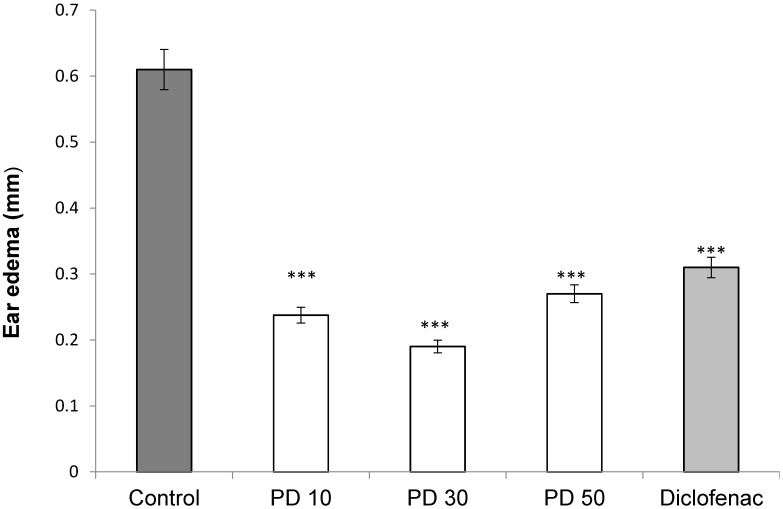
The effect of *P. dermoporus* polysaccharides on the croton oil-induced ear edema assay in BALBc mice. The animals were treated with 10 mg/kg (PD 10), 30 mg/kg (PD 30) and 50 mg/kg (PD 50) of *P. dermoporus* polysaccharides. Data obtained from animal experiments are expressed as the mean ± SD. The differences between treatment and control were tested by ANOVA. A value of (***) *p* < 0.001 was considered statistically significant.

The anti-edematogenic activity of the extract from *P. dermoporus* against croton oil-induced ear edema in rats was assessed. For comparison, the effect of nonsteroidal (indomethacin) anti-inflammatory drug was analyzed. The animals received a subcutaneous injection of croton oil (0.1 mL of 1%) solution in acetone. The ear volume was measured in the groups: positive control rats that received croton oil, saline (negative control) and extracts of *P. dermoporus* (10, 30, and 50 mg/kg). The extract of *P. dermoporus* reduced the edema by 65.6% at 30 mg/kg. At 10 mg/kg, the reduction was 58.3%, and at a concentration of 50 mg/kg, the reduction was 55.7% (Figure 5).

### 3.8. Histological Analysis

**Figure 6:**
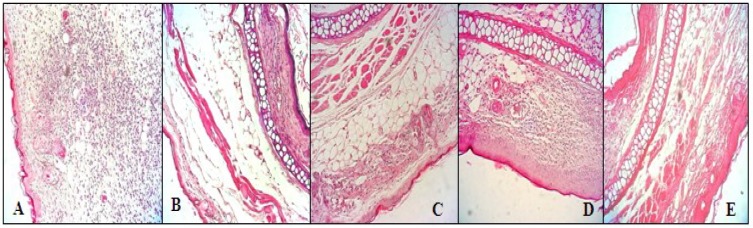
Histological analysis of ear edema with H & E stain 200× from animals submitted to the croton oil-induced ear edema test and treated with *P. dermoporus* polysaccharides: (**A**) positive control (croton oil); (**B**) negative control (saline); (**C**) *P. dermoporus* polysaccharides at 10 mg/kg (PD 10); (**D**) animals treated with 30 mg/kg (PD 30); (**E**) animals treated with 50 mg/kg (PD 50).

To evaluate histological changes, we carried out hematoxylin and eosin (H & E) staining, 200×. Histological examination of ear edema sections (Figure 6A) revealed significant tissue damage to animals treated with croton oil. The slide of animals treated (positive control) presented intense cellular infiltrate, predominantly neutrophilic inflammatory infiltrate (Figure 6A), characteristic of inflammatory reaction. Exposure to saline (Figure 6B) did not lead to significant pathologic alterations. The ear sections of the animals treated with the glucans (Figure 6C–E) were similar to those that received only saline (Figure 6B), showing decreased congestion and infiltration of leukocytes, especially neutrophils into paw tissues.

### 3.9. Cytotoxic Test

The cytotoxicity tests are based on the capacity of the cells to convert to tetrazolium (MTT) in a mixture of blue coloration called formazan [21]. However, only live cells have this capacity. The evaluation of the transformation of MTT in formazan in mononuclear cells of the peripheral blood is accomplished by ELISA, reading at 540 and 620 nm. The cells incubated with the extracts of *P. dermoporus* showed no quantitative difference in relation to the control (*p* < 0.001). Thus, this polysaccharide had no cytotoxic effect.

## 4. Conclusions

The results obtained in this paper showed that glucans of *Polyporus dermoporus* have inhibitory action in the formation of superoxide and hydroxyl radicals. The effect against this radical is dose dependent and inversely proportional to the increase in concentration. In this study, the intrapleural administration of carrageenan produced an acute inflammatory response. The volume and number of cells in pleural fluid increased in the initial 12 h, followed by a decline. A significant decrease in cell numbers was observed in the pleural fluid compared with the control. The value of nitrate/nitrite produced in the pleural cavity of rats by carrageenan administration and previous treatments of *P. dermoporus* extracts at a concentration of 30 mg/kg (PD 30) showed a 69.5% NO decrease (*p* < 0.001) in relation to the control group (carrageenan). This decrease suggests an anti-inflammatory effect of the *P. dermoporus* extract. Overall, this polysaccharide possessed good antioxidant properties and showed anti-inflammatory activity that is not often studied in mushrooms. We suggest that glucans appear to be related to the inhibition of diapedesis after cell migration to the injury site. Analyses conducted in this study demonstrated that the polysaccharide from *P. dermoporus* has no cytotoxic effect.
